# Poly[[μ_4_-naphthalene-1,4-dicarboxyl­ato-κ^4^ *O*:*O*′:*O*′′:*O*′′′-μ_2_-naphthalene-1,4-dicarboxyl­ato-κ^4^ *O*,*O*′:*O*′′,*O*′′′-bis­(2-phenyl-1*H*-1,3,7,8-tetra­azacyclopenta­[*l*]phenanthrene-κ^2^ *N* ^7^,*N* ^8^)dimanganese(II)] *N*,*N*-dimethyl­formamide solvate]

**DOI:** 10.1107/S1600536808010787

**Published:** 2008-04-23

**Authors:** Heng-Da Li, Yang Liu, Mao-Liang Xu, Seik Weng Ng

**Affiliations:** a Department of Chemistry, Jilin Normal University, Siping 136000, People’s Republic of China; b Xi’an Modern Chemistry Research Institute, Xi’an 710065, People’s Republic of China; c Department of Chemistry, University of Malaya, 50603 Kuala Lumpur, Malaysia

## Abstract

One of the two 1,4-dicarboxyl­ate dianions in the title compound, [Mn_2_(C_12_H_6_O_4_)_2_(C_19_H_12_N_4_)_2_]·C_3_H_7_NO, uses its two carboxyl­ate groups to chelate two *N*-heterocycle-chelated Mn atoms; the other 1,4-dicarboxyl­ate dianion binds to four such metal centers. The octa­hedrally coordinated Mn atoms are linked through the two dianions into a layer motif; the dimethyl­formamide mol­ecules occupy the spaces between adjacent layers. Ten C atoms and attached H atoms of one dianion are disordered equally over two positions.

## Related literature

There are several studies of 2-phenyl-1*H*-1,3,7,8-tetra­aza­cyclo­penta­[*l*]phenanthrene-chelated metal compounds; for the structures of the manganese dicarboxyl­ate adducts, see: Che (2006 ▶); Che & Liu (2006 ▶); Wang *et al.* (2006 ▶); Zhang *et al.* (2006 ▶).
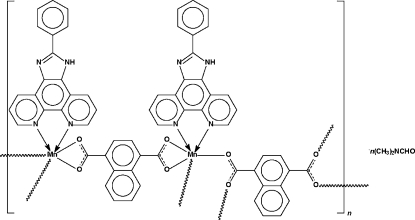


## Experimental

### 

#### Crystal data

[Mn_2_(C_12_H_6_O_4_)_2_(C_19_H_12_N_4_)_2_]·C_3_H_7_NO
*M* *_r_* = 1203.96
Triclinic, 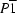

*a* = 9.240 (2) Å
*b* = 14.852 (5) Å
*c* = 21.921 (5) Å
α = 105.72 (1)°
β = 100.48 (1)°
γ = 101.67 (1)°
*V* = 2745.0 (13) Å^3^
*Z* = 2
Mo *K*α radiation
μ = 0.53 mm^−1^
*T* = 295 (2) K
0.26 × 0.16 × 0.12 mm

#### Data collection

Rigaku R-AXIS RAPID diffractometer
Absorption correction: multi-scan (*ABSCOR*; Higashi, 1995 ▶) *T*_min_ = 0.760, *T*_max_ = 0.940
21501 measured reflections
9443 independent reflections
4800 reflections with *I* > 2σ(*I*)
*R*_int_ = 0.084

#### Refinement

*R*[*F* ^2^ > 2σ(*F* ^2^)] = 0.080
*wR*(*F* ^2^) = 0.262
*S* = 1.06
9443 reflections
810 parameters
158 restraints
H-atom parameters constrained
Δρ_max_ = 1.74 e Å^−3^
Δρ_min_ = −0.48 e Å^−3^

### 

Data collection: *RAPID-AUTO* (Rigaku, 1998 ▶); cell refinement: *RAPID-AUTO*; data reduction: *CrystalStructure* (Rigaku/MSC, 2002 ▶); program(s) used to solve structure: *SHELXS97* (Sheldrick, 2008 ▶); program(s) used to refine structure: *SHELXL97* (Sheldrick, 2008 ▶); molecular graphics: *X-SEED* (Barbour, 2001 ▶) and *OLEX* (Dolomanov *et al.*, 2003 ▶); software used to prepare material for publication: *publCIF* (Westrip, 2008 ▶).

## Supplementary Material

Crystal structure: contains datablocks global, I. DOI: 10.1107/S1600536808010787/rz2195sup1.cif

Structure factors: contains datablocks I. DOI: 10.1107/S1600536808010787/rz2195Isup2.hkl

Additional supplementary materials:  crystallographic information; 3D view; checkCIF report

## Figures and Tables

**Table 1:** Selected bond lengths (Å)

Mn1—O1^i^	2.115 (5)
Mn1—O4^ii^	2.105 (4)
Mn1—O5	2.431 (5)
Mn1—O6	2.171 (5)
Mn1—N1	2.232 (5)
Mn1—N4	2.259 (5)
Mn2—O2	2.107 (5)
Mn2—O3^iii^	2.112 (5)
Mn2—O7	2.208 (5)
Mn2—O8	2.384 (5)
Mn2—N5	2.252 (6)
Mn2—N8	2.277 (5)

Symmetry codes: (i) 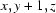; (ii) 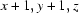; (iii) 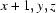.

**Fig. 1.**
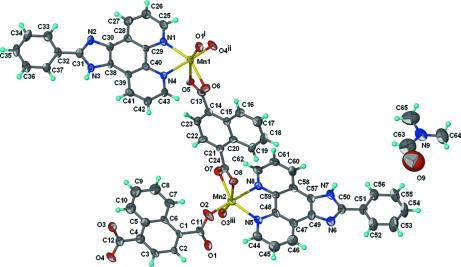
Thermal ellipsoid plot of Mn2(C19H12N4)2(C12H6O4)2.DMF; displacement ellipsoids are drawn at the 50% probability level, and H atoms as spheres of arbitrary radius. The disorder in one of the naphthalene-1,4-dicarboxylate dianions is not shown. Symmetry codes are given in Table 1.

**Fig. 2.**
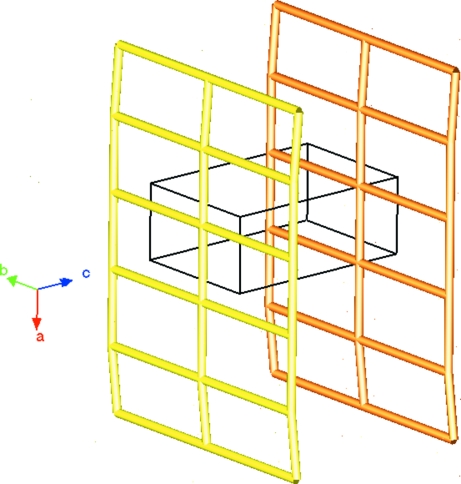
Layer structure of the manganese-naphthalene-1,4-dicarboxylate network as illustrated by OLEX (Dolomanov et al., 2003).

## supplementary crystallographic information

### Comment

There are several studies of metal complexes of 2-phenyl-1*H*-1,3,7,8-tetraazacyclopenta[*l*]phenanthrene. Among these are several manganese complexes, the manganese succinate (Zhang *et al.*, 2006), manganese adipate (Wang *et al.*, 2006), manganese isophthalate (Che & Liu, 2006) and manganese terephthalate (Che, 2006) adducts, all of which feature carboxylate-bridged chain motifs. The title naphthalene-1,4-dicarboxylate adduct adopts a layer motif instead.

One of the two 1,4-dicarboxylate dianions in Mn_2_(C_19_H_12_N_4_)_2_(C_12_H_6_O_4_)_2_^.^DMF uses its two carboxyl –CO_2_ groups to chelate to two *N*-heterocycle-chelated manganese atoms; the other 1,4-dicarboxylate dianion binds to four such metal centers. The octahedrally coordinated manganese atoms are linked through the two dianions into a layer motif; the DMF molecules occupy the spaces between adjacent layers.

### Experimental

Manganese dichloride dihydrate (0.1 mmol), naphthalene-1,4-dicarboxylic acid (0.1 mmol), 2-phenyl-1*H*-1,3,7,8-tetraazacyclopenta[*l*]phenanthrene (0.1 mmol), water (8 ml) and DMF (4 ml) were heated in a 23 ml, Teflon-lined, stainless-steel Parr bomb at 408 K for 2 days. Crystals were obtained in 40% yield.

### Refinement

The naphthalene-1,4-dicarboxylate dianion that is involved in µ_4_-bridging is disordered over two positions in the fused-ring portion. This was refined as two rigid naphthalene groups of 1.39 Å sides; the occupancy was arbitrarily set as 0.5 as this could not be refined. The C1–C11 and C1'–C11 distances were restrained to within 0.01 Å of each other, as were the C4–C12 and C4'–C12 pair of distances. The anisotropic displacement factors of the two fused-rings were restrained to be nearly isotropic.

The carbon-bound H atoms were placed in calculated positions [C–H 0.93, N–H 0.86 Å and *U*_iso_(H) 1.2*U*_eq_(C,*N*)], and were included in the refinement in the riding-model approximation.

The final difference Fourier map had a large peak at 2.8 Å from O7, but this could not be modeled as a water molecule.

### Crystal data

**Table:**

[Mn_2_(C_12_H_6_O_4_)_2_(C_19_H_12_N_4_)_2_]·C_3_H_7_NO	*Z* = 2
*M**_r_* = 1203.96	*F*_000_ = 1236
Triclinic, *P*1	*D*_x_ = 1.457 Mg m^−^^3^
Hall symbol: -P 1	Mo *K*α radiation λ = 0.71073 Å
*a* = 9.240 (2) Å	Cell parameters from 15789 reflections
*b* = 14.852 (5) Å	θ = 3.0–27.5º
*c* = 21.921 (5) Å	µ = 0.53 mm^−^^1^
α = 105.72 (1)º	*T* = 295 (2) K
β = 100.48 (1)º	Block, colorless
γ = 101.67 (1)º	0.26 × 0.16 × 0.12 mm
*V* = 2745.0 (13) Å^3^	

### Data collection

**Table:**

Rigaku R-AXIS RAPID diffractometer	9443 independent reflections
Radiation source: fine-focus sealed tube	4800 reflections with *I* > 2σ(*I*)
Monochromator: graphite	*R*_int_ = 0.084
Detector resolution: 10 pixels mm^-1^	θ_max_ = 25.0º
*T* = 295(2) K	θ_min_ = 3.0º
ω scans	*h* = −10→10
Absorption correction: Multi-scan(ABSCOR; Higashi, 1995)	*k* = −17→17
*T*_min_ = 0.760, *T*_max_ = 0.940	*l* = −26→26
21501 measured reflections	

### Refinement

**Table:**

Refinement on *F*^2^	Secondary atom site location: difference Fourier map
Least-squares matrix: full	Hydrogen site location: inferred from neighbouring sites
*R*[*F*^2^ > 2σ(*F*^2^)] = 0.080	H-atom parameters constrained
*wR*(*F*^2^) = 0.262	*w* = 1/[σ^2^(*F*_o_^2^) + (0.1308*P*)^2^ + 1.0767*P*] where *P* = (*F*_o_^2^ + 2*F*_c_^2^)/3
*S* = 1.06	(Δ/σ)_max_ = 0.001
9443 reflections	Δρ_max_ = 1.74 e Å^−^^3^
810 parameters	Δρ_min_ = −0.48 e Å^−^^3^
158 restraints	Extinction correction: none
Primary atom site location: structure-invariant direct methods	

### Fractional atomic coordinates and isotropic or equivalent isotropic displacement parameters (Å^2^)

**Table:**

	*x*	*y*	*z*	*U*_iso_*/*U*_eq_	Occ. (<1)
Mn1	0.80675 (10)	0.75810 (7)	0.69248 (5)	0.0344 (3)	
Mn2	1.06512 (11)	0.08617 (8)	0.75021 (6)	0.0431 (3)	
O1	0.8244 (5)	−0.1078 (3)	0.7625 (2)	0.0484 (13)	
O2	0.8416 (5)	0.0418 (4)	0.7602 (3)	0.0721 (19)	
O3	0.0485 (5)	−0.0465 (4)	0.6792 (3)	0.0523 (14)	
O4	0.0263 (5)	−0.1993 (4)	0.6783 (3)	0.0539 (14)	
O5	0.8422 (5)	0.5979 (3)	0.6470 (2)	0.0394 (11)	
O6	0.8715 (5)	0.6725 (4)	0.7537 (2)	0.0464 (13)	
O7	1.0143 (5)	0.1880 (4)	0.6976 (3)	0.0500 (13)	
O8	1.0396 (5)	0.2427 (4)	0.8038 (2)	0.0445 (12)	
N1	0.6616 (5)	0.7650 (4)	0.6015 (3)	0.0358 (13)	
N2	0.1203 (6)	0.6871 (5)	0.4819 (3)	0.0418 (15)	
N3	0.0391 (6)	0.6310 (4)	0.5578 (3)	0.0372 (14)	
H3N	−0.0221	0.6062	0.5778	0.045*	
N4	0.5621 (6)	0.6932 (4)	0.6907 (3)	0.0335 (13)	
N5	1.2081 (6)	0.0765 (4)	0.8419 (3)	0.0433 (15)	
N6	1.7453 (6)	0.1593 (5)	0.9659 (3)	0.0479 (16)	
N7	1.8308 (6)	0.2076 (4)	0.8873 (3)	0.0439 (15)	
H7N	1.8936	0.2302	0.8671	0.053*	
N8	1.3130 (6)	0.1489 (4)	0.7526 (3)	0.0427 (15)	
C1	0.5938 (5)	−0.0627 (9)	0.7341 (5)	0.027 (8)	0.50
C2	0.5072 (7)	−0.1025 (11)	0.7707 (5)	0.029 (6)	0.50
H2	0.5551	−0.1156	0.8072	0.035*	0.50
C3	0.3489 (7)	−0.1228 (11)	0.7526 (5)	0.037 (5)	0.50
H3	0.2910	−0.1494	0.7771	0.045*	0.50
C4	0.2773 (5)	−0.1033 (10)	0.6980 (5)	0.048 (8)	0.50
C5	0.3640 (5)	−0.0635 (7)	0.6615 (4)	0.048 (10)	0.50
C6	0.5223 (5)	−0.0432 (7)	0.6795 (4)	0.034 (10)	0.50
C7	0.6089 (7)	−0.0034 (8)	0.6429 (5)	0.054 (4)	0.50
H7	0.7148	0.0102	0.6550	0.064*	0.50
C8	0.5373 (11)	0.0161 (8)	0.5883 (4)	0.071 (5)	0.50
H8	0.5953	0.0428	0.5639	0.085*	0.50
C9	0.3791 (11)	−0.0041 (8)	0.5703 (4)	0.067 (5)	0.50
H9	0.3312	0.0089	0.5338	0.081*	0.50
C10	0.2924 (8)	−0.0439 (9)	0.6069 (4)	0.063 (5)	0.50
H10	0.1865	−0.0575	0.5948	0.076*	0.50
C1'	0.5934 (5)	−0.0562 (9)	0.7394 (4)	0.047 (11)	0.50
C2'	0.5185 (7)	−0.0413 (10)	0.6835 (5)	0.048 (13)	0.50
H2'	0.5741	−0.0190	0.6567	0.058*	0.50
C3'	0.3604 (7)	−0.0597 (9)	0.6676 (4)	0.029 (8)	0.50
H3'	0.3102	−0.0497	0.6301	0.035*	0.50
C4'	0.2772 (5)	−0.0931 (8)	0.7076 (4)	0.021 (5)	0.50
C5'	0.3521 (5)	−0.1080 (5)	0.7635 (3)	0.028 (5)	0.50
C6'	0.5103 (5)	−0.0895 (6)	0.7794 (3)	0.040 (8)	0.50
C7'	0.5852 (6)	−0.1045 (7)	0.8353 (4)	0.039 (3)	0.50
H7'	0.6910	−0.0921	0.8460	0.047*	0.50
C8'	0.5020 (9)	−0.1378 (6)	0.8753 (3)	0.044 (4)	0.50
H8'	0.5522	−0.1478	0.9128	0.053*	0.50
C9'	0.3439 (9)	−0.1562 (6)	0.8594 (4)	0.046 (4)	0.50
H9'	0.2883	−0.1786	0.8862	0.055*	0.50
C10'	0.2690 (6)	−0.1413 (6)	0.8035 (4)	0.042 (3)	0.50
H10'	0.1632	−0.1537	0.7929	0.051*	0.50
C11	0.7665 (7)	−0.0412 (5)	0.7541 (4)	0.0456 (19)	
C12	0.1025 (8)	−0.1168 (6)	0.6854 (3)	0.0422 (18)	
C13	0.8782 (7)	0.6019 (5)	0.7074 (4)	0.0394 (17)	
C14	0.9218 (7)	0.5171 (5)	0.7226 (3)	0.0370 (16)	
C15	1.0253 (7)	0.5249 (5)	0.7819 (3)	0.0346 (16)	
C16	1.0953 (8)	0.6157 (5)	0.8332 (4)	0.0485 (19)	
H16	1.0697	0.6716	0.8290	0.058*	
C17	1.1988 (9)	0.6212 (6)	0.8881 (4)	0.056 (2)	
H17	1.2416	0.6804	0.9212	0.067*	
C18	1.2410 (9)	0.5380 (7)	0.8948 (4)	0.063 (2)	
H18	1.3150	0.5432	0.9315	0.075*	
C19	1.1752 (8)	0.4502 (6)	0.8485 (4)	0.0492 (19)	
H19	1.2030	0.3958	0.8545	0.059*	
C20	1.0646 (7)	0.4397 (5)	0.7909 (3)	0.0369 (16)	
C21	0.9922 (7)	0.3487 (5)	0.7412 (3)	0.0349 (16)	
C22	0.8966 (8)	0.3436 (5)	0.6850 (4)	0.0407 (17)	
H22	0.8517	0.2838	0.6527	0.049*	
C23	0.8646 (7)	0.4275 (5)	0.6751 (3)	0.0388 (17)	
H23	0.8027	0.4226	0.6352	0.047*	
C24	1.0185 (7)	0.2543 (5)	0.7487 (4)	0.0402 (17)	
C25	0.7115 (7)	0.8015 (5)	0.5574 (4)	0.0444 (18)	
H25	0.8161	0.8266	0.5646	0.053*	
C26	0.6146 (8)	0.8038 (6)	0.5007 (4)	0.052 (2)	
H26	0.6547	0.8283	0.4707	0.063*	
C27	0.4607 (8)	0.7695 (6)	0.4904 (3)	0.0472 (19)	
H27	0.3947	0.7705	0.4532	0.057*	
C28	0.4023 (7)	0.7326 (5)	0.5366 (3)	0.0340 (15)	
C29	0.5081 (7)	0.7313 (5)	0.5913 (3)	0.0304 (14)	
C30	0.2444 (7)	0.6954 (5)	0.5310 (3)	0.0360 (16)	
C31	−0.0010 (7)	0.6479 (5)	0.4995 (3)	0.0404 (17)	
C32	−0.1577 (7)	0.6204 (5)	0.4597 (3)	0.0407 (17)	
C33	−0.1849 (8)	0.6449 (6)	0.4017 (4)	0.054 (2)	
H33	−0.1045	0.6802	0.3906	0.065*	
C34	−0.3294 (9)	0.6169 (7)	0.3616 (4)	0.062 (2)	
H34	−0.3451	0.6322	0.3229	0.074*	
C35	−0.4512 (8)	0.5666 (6)	0.3772 (4)	0.057 (2)	
H35	−0.5489	0.5482	0.3498	0.068*	
C36	−0.4247 (8)	0.5435 (6)	0.4359 (4)	0.058 (2)	
H36	−0.5062	0.5104	0.4477	0.070*	
C37	−0.2806 (7)	0.5693 (6)	0.4757 (4)	0.0454 (18)	
H37	−0.2649	0.5525	0.5138	0.055*	
C38	0.1968 (6)	0.6614 (5)	0.5786 (3)	0.0330 (15)	
C39	0.2984 (7)	0.6593 (5)	0.6347 (3)	0.0324 (15)	
C40	0.4544 (7)	0.6934 (5)	0.6397 (3)	0.0344 (15)	
C41	0.2561 (8)	0.6218 (5)	0.6840 (4)	0.0447 (18)	
H41	0.1537	0.5966	0.6814	0.054*	
C42	0.3656 (7)	0.6229 (6)	0.7346 (3)	0.0441 (18)	
H42	0.3389	0.6003	0.7678	0.053*	
C43	0.5183 (7)	0.6582 (5)	0.7367 (3)	0.0406 (17)	
H43	0.5928	0.6574	0.7712	0.049*	
C44	1.1542 (8)	0.0416 (6)	0.8854 (4)	0.059 (2)	
H44	1.0494	0.0165	0.8777	0.070*	
C45	1.2488 (10)	0.0416 (7)	0.9412 (4)	0.072 (3)	
H45	1.2068	0.0186	0.9714	0.086*	
C46	1.4035 (9)	0.0746 (7)	0.9535 (4)	0.064 (2)	
H46	1.4675	0.0727	0.9909	0.076*	
C47	1.4632 (7)	0.1115 (5)	0.9083 (4)	0.0427 (18)	
C48	1.3611 (7)	0.1117 (5)	0.8531 (3)	0.0385 (17)	
C49	1.6241 (7)	0.1468 (5)	0.9147 (4)	0.0435 (18)	
C50	1.8678 (8)	0.1949 (5)	0.9482 (3)	0.0425 (18)	
C51	2.0230 (8)	0.2242 (6)	0.9890 (4)	0.0481 (19)	
C52	2.0480 (9)	0.2083 (7)	1.0480 (4)	0.067 (3)	
H52	1.9674	0.1757	1.0606	0.081*	
C53	2.1936 (11)	0.2406 (8)	1.0891 (4)	0.085 (3)	
H53	2.2091	0.2319	1.1300	0.103*	
C54	2.3144 (10)	0.2850 (8)	1.0708 (5)	0.079 (3)	
H54	2.4119	0.3047	1.0985	0.095*	
C55	2.2923 (9)	0.3005 (7)	1.0116 (5)	0.075 (3)	
H55	2.3744	0.3301	0.9985	0.090*	
C56	2.1443 (9)	0.2713 (7)	0.9708 (4)	0.063 (2)	
H56	2.1278	0.2839	0.9311	0.075*	
C57	1.6738 (7)	0.1767 (5)	0.8654 (3)	0.0386 (17)	
C58	1.5753 (8)	0.1778 (5)	0.8082 (4)	0.0420 (17)	
C59	1.4184 (7)	0.1474 (5)	0.8023 (3)	0.0336 (15)	
C60	1.6205 (8)	0.2092 (6)	0.7593 (4)	0.055 (2)	
H60	1.7237	0.2287	0.7609	0.066*	
C61	1.5131 (9)	0.2115 (6)	0.7086 (4)	0.058 (2)	
H61	1.5414	0.2336	0.6755	0.070*	
C62	1.3589 (8)	0.1797 (6)	0.7075 (4)	0.053 (2)	
H62	1.2856	0.1806	0.6726	0.064*	
O9	2.5478 (19)	0.3811 (10)	0.9394 (7)	0.251 (7)	
N9	2.6817 (9)	0.4487 (5)	0.8833 (4)	0.081 (2)	
C63	2.5473 (15)	0.4057 (7)	0.8923 (7)	0.148 (6)	
H63	2.4561	0.3956	0.8620	0.178*	
C64	2.8345 (11)	0.4717 (9)	0.9235 (6)	0.120 (4)	
H64A	2.8304	0.4595	0.9641	0.181*	
H64B	2.8841	0.5388	0.9322	0.181*	
H64C	2.8908	0.4320	0.9010	0.181*	
C65	2.6539 (17)	0.4727 (9)	0.8232 (5)	0.136 (5)	
H65A	2.5459	0.4589	0.8055	0.204*	
H65B	2.6969	0.4346	0.7921	0.204*	
H65C	2.7004	0.5404	0.8321	0.204*	

### Atomic displacement parameters (Å^2^)

**Table:**

	*U*^11^	*U*^22^	*U*^33^	*U*^12^	*U*^13^	*U*^23^
Mn1	0.0223 (5)	0.0271 (6)	0.0533 (7)	0.0068 (4)	0.0067 (5)	0.0140 (5)
Mn2	0.0265 (6)	0.0289 (7)	0.0766 (8)	0.0103 (5)	0.0118 (5)	0.0196 (6)
O1	0.033 (3)	0.033 (3)	0.070 (3)	0.012 (2)	0.003 (2)	0.006 (3)
O2	0.029 (3)	0.034 (3)	0.151 (6)	0.009 (3)	0.035 (3)	0.016 (4)
O3	0.035 (3)	0.038 (3)	0.083 (4)	0.016 (2)	0.008 (3)	0.019 (3)
O4	0.029 (3)	0.031 (3)	0.101 (4)	0.007 (2)	0.025 (3)	0.016 (3)
O5	0.037 (3)	0.040 (3)	0.046 (3)	0.011 (2)	0.008 (2)	0.024 (2)
O6	0.051 (3)	0.040 (3)	0.059 (3)	0.025 (3)	0.017 (3)	0.020 (3)
O7	0.052 (3)	0.033 (3)	0.066 (3)	0.020 (3)	0.009 (3)	0.016 (3)
O8	0.042 (3)	0.042 (3)	0.063 (3)	0.018 (2)	0.016 (3)	0.030 (3)
N1	0.026 (3)	0.035 (4)	0.044 (3)	0.005 (2)	0.009 (3)	0.012 (3)
N2	0.027 (3)	0.060 (4)	0.037 (3)	0.008 (3)	0.004 (3)	0.018 (3)
N3	0.023 (3)	0.048 (4)	0.040 (3)	0.007 (3)	0.007 (3)	0.017 (3)
N4	0.029 (3)	0.027 (3)	0.038 (3)	0.005 (2)	0.000 (3)	0.008 (3)
N5	0.032 (3)	0.031 (4)	0.066 (4)	0.006 (3)	0.013 (3)	0.014 (3)
N6	0.040 (3)	0.051 (4)	0.053 (4)	0.013 (3)	0.013 (3)	0.014 (3)
N7	0.030 (3)	0.038 (4)	0.058 (4)	0.006 (3)	0.010 (3)	0.007 (3)
N8	0.028 (3)	0.038 (4)	0.065 (4)	0.009 (3)	0.011 (3)	0.021 (3)
C1	0.015 (10)	0.023 (10)	0.042 (10)	0.006 (7)	0.014 (7)	0.003 (6)
C2	0.026 (8)	0.017 (7)	0.036 (8)	0.004 (6)	0.000 (6)	0.002 (6)
C3	0.034 (9)	0.019 (7)	0.049 (8)	0.001 (6)	0.013 (7)	−0.003 (6)
C4	0.050 (11)	0.036 (11)	0.053 (9)	0.012 (8)	0.008 (8)	0.008 (7)
C5	0.045 (13)	0.047 (13)	0.055 (12)	0.013 (8)	0.013 (9)	0.020 (8)
C6	0.023 (12)	0.030 (13)	0.048 (12)	0.002 (7)	0.018 (8)	0.011 (7)
C7	0.053 (7)	0.049 (8)	0.061 (8)	0.011 (6)	0.018 (6)	0.021 (6)
C8	0.068 (8)	0.076 (9)	0.073 (8)	0.016 (7)	0.018 (7)	0.032 (7)
C9	0.059 (8)	0.076 (9)	0.075 (8)	0.027 (7)	0.019 (7)	0.030 (7)
C10	0.054 (8)	0.070 (9)	0.069 (8)	0.019 (7)	0.015 (7)	0.026 (7)
C1'	0.038 (13)	0.035 (13)	0.060 (13)	0.010 (9)	0.008 (9)	0.004 (8)
C2'	0.039 (15)	0.043 (15)	0.061 (14)	0.011 (9)	0.016 (9)	0.011 (8)
C3'	0.015 (9)	0.029 (10)	0.044 (10)	0.015 (7)	0.005 (7)	0.009 (6)
C4'	0.010 (7)	0.008 (7)	0.045 (8)	0.005 (5)	0.013 (6)	0.003 (5)
C5'	0.025 (8)	0.016 (8)	0.040 (8)	0.010 (6)	0.007 (6)	0.001 (6)
C6'	0.043 (10)	0.034 (10)	0.047 (10)	0.013 (7)	0.017 (8)	0.011 (7)
C7'	0.038 (6)	0.034 (7)	0.045 (7)	0.015 (5)	0.006 (5)	0.009 (5)
C8'	0.041 (6)	0.042 (7)	0.051 (7)	0.015 (6)	0.010 (6)	0.015 (6)
C9'	0.054 (7)	0.035 (7)	0.053 (7)	0.009 (6)	0.026 (6)	0.014 (6)
C10'	0.037 (6)	0.036 (7)	0.054 (7)	0.013 (5)	0.017 (6)	0.008 (6)
C11	0.026 (3)	0.029 (4)	0.075 (5)	0.004 (3)	0.012 (4)	0.009 (4)
C12	0.035 (4)	0.043 (5)	0.047 (4)	0.012 (4)	0.011 (3)	0.010 (4)
C13	0.028 (3)	0.036 (4)	0.063 (5)	0.008 (3)	0.019 (4)	0.024 (4)
C14	0.032 (4)	0.032 (4)	0.052 (4)	0.013 (3)	0.016 (3)	0.014 (3)
C15	0.028 (3)	0.040 (4)	0.040 (4)	0.010 (3)	0.011 (3)	0.015 (3)
C16	0.052 (5)	0.033 (5)	0.059 (5)	0.017 (4)	0.009 (4)	0.011 (4)
C17	0.062 (5)	0.042 (5)	0.052 (5)	0.014 (4)	0.004 (4)	0.004 (4)
C18	0.045 (5)	0.070 (7)	0.061 (5)	0.010 (5)	−0.008 (4)	0.020 (5)
C19	0.041 (4)	0.047 (5)	0.057 (5)	0.015 (4)	−0.004 (4)	0.021 (4)
C20	0.033 (3)	0.035 (4)	0.053 (4)	0.014 (3)	0.017 (3)	0.022 (4)
C21	0.030 (3)	0.030 (4)	0.048 (4)	0.009 (3)	0.013 (3)	0.015 (3)
C22	0.047 (4)	0.028 (4)	0.052 (4)	0.016 (3)	0.013 (4)	0.017 (3)
C23	0.029 (3)	0.050 (5)	0.040 (4)	0.012 (3)	0.008 (3)	0.018 (4)
C24	0.029 (4)	0.033 (4)	0.065 (5)	0.015 (3)	0.014 (4)	0.021 (4)
C25	0.027 (3)	0.050 (5)	0.060 (5)	0.008 (3)	0.019 (4)	0.019 (4)
C26	0.047 (4)	0.063 (6)	0.050 (5)	0.011 (4)	0.015 (4)	0.025 (4)
C27	0.049 (4)	0.060 (6)	0.039 (4)	0.018 (4)	0.013 (4)	0.023 (4)
C28	0.031 (3)	0.034 (4)	0.038 (4)	0.009 (3)	0.012 (3)	0.011 (3)
C29	0.025 (3)	0.028 (4)	0.036 (4)	0.004 (3)	0.005 (3)	0.011 (3)
C30	0.030 (4)	0.041 (4)	0.033 (4)	0.009 (3)	0.007 (3)	0.008 (3)
C31	0.029 (4)	0.048 (5)	0.041 (4)	0.013 (3)	0.008 (3)	0.010 (4)
C32	0.030 (4)	0.044 (5)	0.040 (4)	0.010 (3)	0.005 (3)	0.003 (3)
C33	0.042 (4)	0.064 (6)	0.057 (5)	0.011 (4)	0.011 (4)	0.023 (4)
C34	0.045 (5)	0.088 (7)	0.057 (5)	0.029 (5)	−0.001 (4)	0.030 (5)
C35	0.027 (4)	0.065 (6)	0.067 (5)	0.016 (4)	−0.001 (4)	0.010 (5)
C36	0.037 (4)	0.060 (6)	0.069 (6)	0.007 (4)	0.009 (4)	0.013 (5)
C37	0.034 (4)	0.049 (5)	0.052 (4)	0.009 (3)	0.007 (4)	0.019 (4)
C38	0.020 (3)	0.042 (4)	0.036 (4)	0.011 (3)	0.002 (3)	0.011 (3)
C39	0.026 (3)	0.027 (4)	0.043 (4)	0.007 (3)	0.007 (3)	0.009 (3)
C40	0.034 (4)	0.023 (4)	0.039 (4)	0.003 (3)	0.005 (3)	0.003 (3)
C41	0.033 (4)	0.048 (5)	0.060 (5)	0.007 (3)	0.019 (4)	0.026 (4)
C42	0.036 (4)	0.060 (5)	0.040 (4)	0.009 (4)	0.008 (3)	0.024 (4)
C43	0.027 (3)	0.051 (5)	0.042 (4)	0.007 (3)	0.002 (3)	0.019 (4)
C44	0.033 (4)	0.066 (6)	0.085 (6)	0.009 (4)	0.021 (5)	0.037 (5)
C45	0.056 (5)	0.102 (8)	0.075 (6)	0.018 (5)	0.032 (5)	0.049 (6)
C46	0.056 (5)	0.084 (7)	0.062 (5)	0.020 (5)	0.021 (5)	0.033 (5)
C47	0.032 (4)	0.041 (5)	0.056 (5)	0.007 (3)	0.017 (4)	0.015 (4)
C48	0.029 (3)	0.024 (4)	0.054 (4)	0.003 (3)	0.011 (3)	0.001 (3)
C49	0.033 (4)	0.042 (5)	0.051 (4)	0.016 (3)	0.005 (4)	0.007 (4)
C50	0.034 (4)	0.045 (5)	0.045 (4)	0.011 (3)	0.009 (3)	0.009 (4)
C51	0.038 (4)	0.053 (5)	0.045 (4)	0.012 (4)	0.006 (4)	0.006 (4)
C52	0.042 (5)	0.090 (8)	0.057 (5)	0.010 (5)	0.008 (4)	0.013 (5)
C53	0.066 (6)	0.131 (10)	0.034 (5)	0.015 (6)	−0.004 (5)	0.006 (5)
C54	0.037 (5)	0.105 (9)	0.070 (7)	0.008 (5)	−0.006 (5)	0.010 (6)
C55	0.039 (5)	0.084 (8)	0.084 (7)	0.002 (5)	0.013 (5)	0.011 (6)
C56	0.046 (5)	0.064 (6)	0.067 (6)	0.010 (4)	0.002 (4)	0.015 (5)
C57	0.030 (4)	0.031 (4)	0.053 (4)	0.012 (3)	0.012 (4)	0.008 (3)
C58	0.037 (4)	0.034 (4)	0.057 (5)	0.006 (3)	0.017 (4)	0.015 (4)
C59	0.032 (3)	0.023 (4)	0.044 (4)	0.008 (3)	0.006 (3)	0.009 (3)
C60	0.026 (4)	0.063 (6)	0.078 (6)	0.008 (4)	0.010 (4)	0.031 (5)
C61	0.047 (5)	0.056 (6)	0.082 (6)	0.007 (4)	0.019 (5)	0.041 (5)
C62	0.038 (4)	0.050 (5)	0.077 (6)	0.007 (4)	0.015 (4)	0.032 (5)
O9	0.266 (11)	0.249 (11)	0.242 (10)	0.055 (8)	0.089 (8)	0.080 (8)
N9	0.072 (5)	0.072 (5)	0.096 (5)	0.011 (4)	0.031 (4)	0.019 (4)
C63	0.159 (8)	0.143 (8)	0.145 (8)	0.046 (6)	0.043 (6)	0.044 (6)
C64	0.086 (9)	0.110 (11)	0.142 (11)	0.010 (8)	0.012 (8)	0.027 (9)
C65	0.180 (9)	0.124 (9)	0.122 (8)	0.042 (7)	0.061 (7)	0.052 (7)

### Geometric parameters (Å, °)

**Table:**

Mn1—O1^i^	2.115 (5)	C17—C18	1.406 (11)
Mn1—O4^ii^	2.105 (4)	C17—H17	0.9300
Mn1—O5	2.431 (5)	C18—C19	1.355 (11)
Mn1—O6	2.171 (5)	C18—H18	0.9300
Mn1—N1	2.232 (5)	C19—C20	1.421 (9)
Mn1—N4	2.259 (5)	C19—H19	0.9300
Mn2—O2	2.107 (5)	C20—C21	1.426 (9)
Mn2—O3^iii^	2.112 (5)	C21—C22	1.351 (9)
Mn2—O7	2.208 (5)	C21—C24	1.516 (9)
Mn2—O8	2.384 (5)	C22—C23	1.400 (9)
Mn2—N5	2.252 (6)	C22—H22	0.9300
Mn2—N8	2.277 (5)	C23—H23	0.9300
O1—C11	1.255 (8)	C25—C26	1.407 (10)
O2—C11	1.246 (8)	C25—H25	0.9300
O3—C12	1.272 (8)	C26—C27	1.363 (9)
O4—C12	1.237 (8)	C26—H26	0.9300
O4—Mn1^iv^	2.105 (4)	C27—C28	1.414 (9)
O5—C13	1.285 (8)	C27—H27	0.9300
O6—C13	1.268 (8)	C28—C29	1.410 (9)
O7—C24	1.264 (8)	C28—C30	1.421 (8)
O8—C24	1.252 (8)	C29—C40	1.445 (9)
N1—C25	1.335 (8)	C30—C38	1.378 (9)
N1—C29	1.360 (7)	C31—C32	1.460 (9)
N2—C31	1.325 (8)	C32—C37	1.389 (9)
N2—C30	1.381 (8)	C32—C33	1.408 (10)
N3—C31	1.370 (9)	C33—C34	1.371 (10)
N3—C38	1.384 (7)	C33—H33	0.9300
N3—H3N	0.8600	C34—C35	1.371 (11)
N4—C43	1.342 (8)	C34—H34	0.9300
N4—C40	1.357 (8)	C35—C36	1.411 (11)
N5—C44	1.327 (9)	C35—H35	0.9300
N5—C48	1.356 (8)	C36—C37	1.367 (10)
N6—C50	1.319 (8)	C36—H36	0.9300
N6—C49	1.375 (9)	C37—H37	0.9300
N7—C57	1.380 (8)	C38—C39	1.416 (9)
N7—C50	1.390 (9)	C39—C40	1.401 (8)
N7—H7N	0.8600	C39—C41	1.423 (9)
N8—C62	1.298 (9)	C41—C42	1.353 (9)
N8—C59	1.331 (8)	C41—H41	0.9300
C1—C2	1.3900	C42—C43	1.390 (9)
C1—C6	1.3900	C42—H42	0.9300
C1—C11	1.517 (8)	C43—H43	0.9300
C2—C3	1.3900	C44—C45	1.370 (11)
C2—H2	0.9300	C44—H44	0.9300
C3—C4	1.3900	C45—C46	1.365 (11)
C3—H3	0.9300	C45—H45	0.9300
C4—C5	1.3900	C46—C47	1.399 (10)
C4—C12	1.550 (8)	C46—H46	0.9300
C5—C6	1.3900	C47—C48	1.393 (10)
C5—C10	1.3900	C47—C49	1.441 (9)
C6—C7	1.3900	C48—C59	1.490 (9)
C7—C8	1.3900	C49—C57	1.391 (10)
C7—H7	0.9300	C50—C51	1.454 (10)
C8—C9	1.3900	C51—C52	1.365 (11)
C8—H8	0.9300	C51—C56	1.379 (11)
C9—C10	1.3900	C52—C53	1.384 (11)
C9—H9	0.9300	C52—H52	0.9300
C10—H10	0.9300	C53—C54	1.360 (13)
C1'—C2'	1.3900	C53—H53	0.9300
C1'—C6'	1.3900	C54—C55	1.365 (13)
C1'—C11	1.530 (8)	C54—H54	0.9300
C2'—C3'	1.3900	C55—C56	1.400 (11)
C2'—H2'	0.9300	C55—H55	0.9300
C3'—C4'	1.3900	C56—H56	0.9300
C3'—H3'	0.9300	C57—C58	1.415 (10)
C4'—C5'	1.3900	C58—C60	1.377 (10)
C4'—C12	1.535 (8)	C58—C59	1.399 (9)
C5'—C6'	1.3900	C60—C61	1.362 (11)
C5'—C10'	1.3900	C60—H60	0.9300
C6'—C7'	1.3900	C61—C62	1.401 (10)
C7'—C8'	1.3900	C61—H61	0.9300
C7'—H7'	0.9300	C62—H62	0.9300
C8'—C9'	1.3900	O9—C63	1.18 (2)
C8'—H8'	0.9300	N9—C63	1.351 (9)
C9'—C10'	1.3900	N9—C64	1.440 (8)
C9'—H9'	0.9300	N9—C65	1.449 (8)
C10'—H10'	0.9300	C63—H63	0.9300
C13—C14	1.499 (9)	C64—H64A	0.9600
C14—C23	1.378 (9)	C64—H64B	0.9600
C14—C15	1.427 (9)	C64—H64C	0.9600
C15—C16	1.437 (10)	C65—H65A	0.9600
C15—C20	1.438 (9)	C65—H65B	0.9600
C16—C17	1.366 (10)	C65—H65C	0.9600
C16—H16	0.9300		
			
O4^ii^—Mn1—O1^i^	94.88 (19)	C20—C19—H19	119.3
O4^ii^—Mn1—O6	94.6 (2)	C19—C20—C21	123.1 (6)
O1^i^—Mn1—O6	102.2 (2)	C19—C20—C15	118.5 (7)
O4^ii^—Mn1—N1	101.0 (2)	C21—C20—C15	118.3 (6)
O1^i^—Mn1—N1	103.1 (2)	C22—C21—C20	120.8 (6)
O6—Mn1—N1	148.9 (2)	C22—C21—C24	117.3 (7)
O4^ii^—Mn1—N4	170.2 (2)	C20—C21—C24	121.9 (6)
O1^i^—Mn1—N4	94.20 (18)	C21—C22—C23	120.6 (7)
O6—Mn1—N4	87.09 (19)	C21—C22—H22	119.7
N1—Mn1—N4	73.34 (19)	C23—C22—H22	119.7
O4^ii^—Mn1—O5	81.46 (17)	C14—C23—C22	122.0 (7)
O1^i^—Mn1—O5	159.11 (18)	C14—C23—H23	119.0
O6—Mn1—O5	57.90 (17)	C22—C23—H23	119.0
N1—Mn1—O5	97.75 (18)	O8—C24—O7	121.9 (6)
N4—Mn1—O5	91.31 (17)	O8—C24—C21	120.3 (7)
O2—Mn2—O3^iii^	94.0 (2)	O7—C24—C21	117.8 (7)
O2—Mn2—O7	95.2 (2)	O8—C24—Mn2	65.0 (4)
O3^iii^—Mn2—O7	107.4 (2)	O7—C24—Mn2	56.9 (4)
O2—Mn2—N5	102.3 (2)	C21—C24—Mn2	174.7 (5)
O3^iii^—Mn2—N5	103.1 (2)	N1—C25—C26	123.6 (6)
O7—Mn2—N5	143.5 (2)	N1—C25—H25	118.2
O2—Mn2—N8	172.5 (2)	C26—C25—H25	118.2
O3^iii^—Mn2—N8	93.1 (2)	C27—C26—C25	118.9 (7)
O7—Mn2—N8	85.2 (2)	C27—C26—H26	120.6
N5—Mn2—N8	73.5 (2)	C25—C26—H26	120.6
O2—Mn2—O8	82.28 (18)	C26—C27—C28	119.6 (7)
O3^iii^—Mn2—O8	163.32 (19)	C26—C27—H27	120.2
O7—Mn2—O8	57.07 (18)	C28—C27—H27	120.2
N5—Mn2—O8	93.55 (19)	C29—C28—C27	117.6 (6)
N8—Mn2—O8	91.70 (19)	C29—C28—C30	118.0 (6)
C11—O1—Mn1^v^	127.7 (5)	C27—C28—C30	124.3 (6)
C11—O2—Mn2	128.6 (5)	N1—C29—C28	122.9 (6)
C12—O3—Mn2^vi^	129.6 (5)	N1—C29—C40	117.3 (6)
C12—O4—Mn1^iv^	128.5 (4)	C28—C29—C40	119.8 (5)
C13—O5—Mn1	83.7 (4)	C38—C30—N2	110.4 (5)
C13—O6—Mn1	95.8 (4)	C38—C30—C28	120.7 (6)
C24—O7—Mn2	94.4 (4)	N2—C30—C28	128.9 (6)
C24—O8—Mn2	86.6 (4)	N2—C31—N3	111.7 (6)
C25—N1—C29	117.5 (6)	N2—C31—C32	124.3 (6)
C25—N1—Mn1	126.0 (4)	N3—C31—C32	123.8 (6)
C29—N1—Mn1	116.5 (4)	C37—C32—C33	118.5 (6)
C31—N2—C30	105.4 (5)	C37—C32—C31	123.1 (7)
C31—N3—C38	107.0 (5)	C33—C32—C31	118.5 (6)
C31—N3—H3N	126.5	C34—C33—C32	120.4 (7)
C38—N3—H3N	126.5	C34—C33—H33	119.8
C43—N4—C40	119.2 (5)	C32—C33—H33	119.8
C43—N4—Mn1	124.8 (4)	C33—C34—C35	121.3 (8)
C40—N4—Mn1	115.9 (4)	C33—C34—H34	119.3
C44—N5—C48	118.7 (7)	C35—C34—H34	119.3
C44—N5—Mn2	125.3 (5)	C34—C35—C36	118.4 (7)
C48—N5—Mn2	116.0 (5)	C34—C35—H35	120.8
C50—N6—C49	105.2 (6)	C36—C35—H35	120.8
C57—N7—C50	106.7 (6)	C37—C36—C35	120.8 (7)
C57—N7—H7N	126.7	C37—C36—H36	119.6
C50—N7—H7N	126.7	C35—C36—H36	119.6
C62—N8—C59	118.0 (6)	C36—C37—C32	120.6 (7)
C62—N8—Mn2	125.3 (5)	C36—C37—H37	119.7
C59—N8—Mn2	116.5 (4)	C32—C37—H37	119.7
C2—C1—C6	120.0	C30—C38—N3	105.5 (5)
C2—C1—C11	119.5 (5)	C30—C38—C39	123.4 (6)
C6—C1—C11	120.5 (5)	N3—C38—C39	131.0 (6)
C1—C2—C3	120.0	C40—C39—C38	116.2 (6)
C1—C2—H2	120.0	C40—C39—C41	117.8 (6)
C3—C2—H2	120.0	C38—C39—C41	125.9 (6)
C4—C3—C2	120.0	N4—C40—C39	121.4 (6)
C4—C3—H3	120.0	N4—C40—C29	116.9 (5)
C2—C3—H3	120.0	C39—C40—C29	121.8 (6)
C3—C4—C5	120.0	C42—C41—C39	119.7 (6)
C3—C4—C12	117.1 (5)	C42—C41—H41	120.1
C5—C4—C12	122.6 (5)	C39—C41—H41	120.1
C6—C5—C4	120.0	C41—C42—C43	119.4 (6)
C6—C5—C10	120.0	C41—C42—H42	120.3
C4—C5—C10	120.0	C43—C42—H42	120.3
C7—C6—C5	120.0	N4—C43—C42	122.4 (6)
C7—C6—C1	120.0	N4—C43—H43	118.8
C5—C6—C1	120.0	C42—C43—H43	118.8
C6—C7—C8	120.0	N5—C44—C45	121.8 (7)
C6—C7—H7	120.0	N5—C44—H44	119.1
C8—C7—H7	120.0	C45—C44—H44	119.1
C9—C8—C7	120.0	C46—C45—C44	121.2 (8)
C9—C8—H8	120.0	C46—C45—H45	119.4
C7—C8—H8	120.0	C44—C45—H45	119.4
C8—C9—C10	120.0	C45—C46—C47	118.1 (8)
C8—C9—H9	120.0	C45—C46—H46	120.9
C10—C9—H9	120.0	C47—C46—H46	120.9
C9—C10—C5	120.0	C48—C47—C46	118.1 (7)
C9—C10—H10	120.0	C48—C47—C49	118.2 (6)
C5—C10—H10	120.0	C46—C47—C49	123.7 (7)
C2'—C1'—C6'	120.0	N5—C48—C47	122.1 (6)
C2'—C1'—C11	119.4 (5)	N5—C48—C59	117.4 (6)
C6'—C1'—C11	120.6 (5)	C47—C48—C59	120.4 (6)
C1'—C2'—C3'	120.0	N6—C49—C57	111.1 (6)
C1'—C2'—H2'	120.0	N6—C49—C47	129.0 (7)
C3'—C2'—H2'	120.0	C57—C49—C47	119.9 (6)
C2'—C3'—C4'	120.0	N6—C50—N7	111.9 (6)
C2'—C3'—H3'	120.0	N6—C50—C51	124.6 (7)
C4'—C3'—H3'	120.0	N7—C50—C51	123.3 (6)
C5'—C4'—C3'	120.0	C52—C51—C56	119.3 (7)
C5'—C4'—C12	122.6 (4)	C52—C51—C50	119.0 (7)
C3'—C4'—C12	117.4 (4)	C56—C51—C50	121.7 (7)
C4'—C5'—C6'	120.0	C51—C52—C53	119.8 (8)
C4'—C5'—C10'	120.0	C51—C52—H52	120.1
C6'—C5'—C10'	120.0	C53—C52—H52	120.1
C7'—C6'—C5'	120.0	C54—C53—C52	121.2 (9)
C7'—C6'—C1'	120.0	C54—C53—H53	119.4
C5'—C6'—C1'	120.0	C52—C53—H53	119.4
C8'—C7'—C6'	120.0	C53—C54—C55	119.9 (8)
C8'—C7'—H7'	120.0	C53—C54—H54	120.0
C6'—C7'—H7'	120.0	C55—C54—H54	120.0
C9'—C8'—C7'	120.0	C54—C55—C56	119.2 (8)
C9'—C8'—H8'	120.0	C54—C55—H55	120.4
C7'—C8'—H8'	120.0	C56—C55—H55	120.4
C10'—C9'—C8'	120.0	C51—C56—C55	120.5 (9)
C10'—C9'—H9'	120.0	C51—C56—H56	119.7
C8'—C9'—H9'	120.0	C55—C56—H56	119.7
C9'—C10'—C5'	120.0	N7—C57—C49	105.1 (6)
C9'—C10'—H10'	120.0	N7—C57—C58	130.6 (7)
C5'—C10'—H10'	120.0	C49—C57—C58	124.2 (6)
O2—C11—O1	124.3 (6)	C60—C58—C59	117.5 (7)
O2—C11—C1	117.2 (7)	C60—C58—C57	125.7 (6)
O1—C11—C1	118.5 (7)	C59—C58—C57	116.8 (6)
O2—C11—C1'	114.9 (7)	N8—C59—C58	123.1 (6)
O1—C11—C1'	120.7 (7)	N8—C59—C48	116.4 (6)
O4—C12—O3	125.5 (6)	C58—C59—C48	120.4 (6)
O4—C12—C4'	118.6 (7)	C61—C60—C58	119.6 (7)
O3—C12—C4'	115.8 (7)	C61—C60—H60	120.2
O4—C12—C4	115.2 (7)	C58—C60—H60	120.2
O3—C12—C4	119.2 (8)	C60—C61—C62	118.2 (7)
O6—C13—O5	122.5 (6)	C60—C61—H61	120.9
O6—C13—C14	119.2 (6)	C62—C61—H61	120.9
O5—C13—C14	118.2 (6)	N8—C62—C61	123.5 (8)
O6—C13—Mn1	55.5 (3)	N8—C62—H62	118.2
O5—C13—Mn1	67.1 (4)	C61—C62—H62	118.2
C14—C13—Mn1	174.6 (5)	C63—N9—C64	129.9 (11)
C23—C14—C15	118.5 (6)	C63—N9—C65	109.3 (11)
C23—C14—C13	117.7 (7)	C64—N9—C65	120.8 (10)
C15—C14—C13	123.7 (7)	O9—C63—N9	118.8 (16)
C14—C15—C16	122.5 (6)	O9—C63—H63	120.6
C14—C15—C20	119.6 (6)	N9—C63—H63	120.6
C16—C15—C20	117.9 (6)	N9—C64—H64A	109.5
C17—C16—C15	120.9 (7)	N9—C64—H64B	109.5
C17—C16—H16	119.6	H64A—C64—H64B	109.5
C15—C16—H16	119.6	N9—C64—H64C	109.5
C16—C17—C18	120.4 (8)	H64A—C64—H64C	109.5
C16—C17—H17	119.8	H64B—C64—H64C	109.5
C18—C17—H17	119.8	N9—C65—H65A	109.5
C19—C18—C17	120.8 (8)	N9—C65—H65B	109.5
C19—C18—H18	119.6	H65A—C65—H65B	109.5
C17—C18—H18	119.6	N9—C65—H65C	109.5
C18—C19—C20	121.3 (7)	H65A—C65—H65C	109.5
C18—C19—H19	119.3	H65B—C65—H65C	109.5
			
O3^iii^—Mn2—O2—C11	31.4 (8)	C13—C14—C15—C16	1.2 (9)
O7—Mn2—O2—C11	139.3 (8)	C23—C14—C15—C20	−1.1 (9)
N5—Mn2—O2—C11	−73.0 (8)	C13—C14—C15—C20	−177.6 (5)
O8—Mn2—O2—C11	−165.0 (8)	C14—C15—C16—C17	−177.1 (7)
C24—Mn2—O2—C11	167.4 (8)	C20—C15—C16—C17	1.6 (10)
O4^ii^—Mn1—O5—C13	98.9 (4)	C15—C16—C17—C18	1.2 (12)
O1^i^—Mn1—O5—C13	17.7 (6)	C16—C17—C18—C19	−2.9 (13)
O6—Mn1—O5—C13	−1.9 (3)	C17—C18—C19—C20	1.6 (12)
N1—Mn1—O5—C13	−161.1 (3)	C18—C19—C20—C21	−179.7 (7)
N4—Mn1—O5—C13	−87.7 (4)	C18—C19—C20—C15	1.2 (10)
O4^ii^—Mn1—O6—C13	−75.0 (4)	C14—C15—C20—C19	176.1 (6)
O1^i^—Mn1—O6—C13	−171.0 (4)	C16—C15—C20—C19	−2.7 (9)
N1—Mn1—O6—C13	45.1 (5)	C14—C15—C20—C21	−3.1 (8)
N4—Mn1—O6—C13	95.4 (4)	C16—C15—C20—C21	178.1 (6)
O5—Mn1—O6—C13	2.0 (3)	C19—C20—C21—C22	−174.9 (6)
O2—Mn2—O7—C24	78.8 (4)	C15—C20—C21—C22	4.3 (9)
O3^iii^—Mn2—O7—C24	174.7 (4)	C19—C20—C21—C24	5.1 (9)
N5—Mn2—O7—C24	−39.9 (5)	C15—C20—C21—C24	−175.8 (5)
N8—Mn2—O7—C24	−93.6 (4)	C20—C21—C22—C23	−1.1 (9)
O8—Mn2—O7—C24	1.5 (4)	C24—C21—C22—C23	178.9 (6)
O2—Mn2—O8—C24	−102.9 (4)	C15—C14—C23—C22	4.4 (9)
O3^iii^—Mn2—O8—C24	−24.9 (8)	C13—C14—C23—C22	−178.9 (6)
O7—Mn2—O8—C24	−1.5 (4)	C21—C22—C23—C14	−3.4 (10)
N5—Mn2—O8—C24	155.2 (4)	Mn2—O8—C24—O7	2.6 (6)
N8—Mn2—O8—C24	81.7 (4)	Mn2—O8—C24—C21	−179.3 (6)
O4^ii^—Mn1—N1—C25	−8.7 (6)	Mn2—O7—C24—O8	−2.9 (7)
O1^i^—Mn1—N1—C25	89.0 (6)	Mn2—O7—C24—C21	179.0 (5)
O6—Mn1—N1—C25	−127.2 (6)	C22—C21—C24—O8	−146.0 (6)
N4—Mn1—N1—C25	179.5 (6)	C20—C21—C24—O8	34.0 (9)
O5—Mn1—N1—C25	−91.4 (6)	C22—C21—C24—O7	32.2 (9)
C13—Mn1—N1—C25	−102.6 (6)	C20—C21—C24—O7	−147.8 (6)
O4^ii^—Mn1—N1—C29	173.2 (4)	C29—N1—C25—C26	−2.0 (10)
O1^i^—Mn1—N1—C29	−89.1 (5)	Mn1—N1—C25—C26	179.9 (6)
O6—Mn1—N1—C29	54.7 (6)	N1—C25—C26—C27	1.7 (12)
N4—Mn1—N1—C29	1.4 (4)	C25—C26—C27—C28	0.0 (12)
O5—Mn1—N1—C29	90.5 (5)	C26—C27—C28—C29	−1.2 (11)
C13—Mn1—N1—C29	79.3 (5)	C26—C27—C28—C30	179.8 (7)
O1^i^—Mn1—N4—C43	−77.6 (6)	C25—N1—C29—C28	0.7 (10)
O6—Mn1—N4—C43	24.5 (5)	Mn1—N1—C29—C28	178.9 (5)
N1—Mn1—N4—C43	180.0 (6)	C25—N1—C29—C40	−179.1 (6)
O5—Mn1—N4—C43	82.3 (5)	Mn1—N1—C29—C40	−0.8 (7)
C13—Mn1—N4—C43	53.1 (6)	C27—C28—C29—N1	0.9 (10)
O1^i^—Mn1—N4—C40	100.5 (5)	C30—C28—C29—N1	180.0 (6)
O6—Mn1—N4—C40	−157.4 (5)	C27—C28—C29—C40	−179.4 (6)
N1—Mn1—N4—C40	−1.9 (4)	C30—C28—C29—C40	−0.3 (10)
O5—Mn1—N4—C40	−99.6 (5)	C31—N2—C30—C38	−0.3 (8)
C13—Mn1—N4—C40	−128.8 (5)	C31—N2—C30—C28	179.8 (7)
O2—Mn2—N5—C44	5.6 (7)	C29—C28—C30—C38	1.5 (10)
O3^iii^—Mn2—N5—C44	−91.5 (6)	C27—C28—C30—C38	−179.5 (7)
O7—Mn2—N5—C44	122.3 (6)	C29—C28—C30—N2	−178.6 (7)
N8—Mn2—N5—C44	179.2 (7)	C27—C28—C30—N2	0.4 (12)
O8—Mn2—N5—C44	88.5 (6)	C30—N2—C31—N3	0.3 (8)
C24—Mn2—N5—C44	101.7 (6)	C30—N2—C31—C32	−176.1 (7)
O2—Mn2—N5—C48	−172.7 (5)	C38—N3—C31—N2	−0.1 (8)
O3^iii^—Mn2—N5—C48	90.2 (5)	C38—N3—C31—C32	176.3 (7)
O7—Mn2—N5—C48	−56.0 (6)	N2—C31—C32—C37	172.2 (7)
N8—Mn2—N5—C48	0.9 (5)	N3—C31—C32—C37	−3.8 (11)
O8—Mn2—N5—C48	−89.9 (5)	N2—C31—C32—C33	−6.2 (11)
C24—Mn2—N5—C48	−76.7 (5)	N3—C31—C32—C33	177.8 (7)
O3^iii^—Mn2—N8—C62	74.5 (6)	C37—C32—C33—C34	−1.3 (11)
O7—Mn2—N8—C62	−32.8 (6)	C31—C32—C33—C34	177.2 (7)
N5—Mn2—N8—C62	177.3 (7)	C32—C33—C34—C35	1.5 (13)
O8—Mn2—N8—C62	−89.5 (6)	C33—C34—C35—C36	−0.3 (13)
C24—Mn2—N8—C62	−61.4 (6)	C34—C35—C36—C37	−1.0 (12)
O3^iii^—Mn2—N8—C59	−100.8 (5)	C35—C36—C37—C32	1.2 (12)
O7—Mn2—N8—C59	152.0 (5)	C33—C32—C37—C36	−0.1 (11)
N5—Mn2—N8—C59	2.0 (5)	C31—C32—C37—C36	−178.5 (7)
O8—Mn2—N8—C59	95.2 (5)	N2—C30—C38—N3	0.3 (8)
C24—Mn2—N8—C59	123.4 (5)	C28—C30—C38—N3	−179.8 (6)
C6—C1—C2—C3	0.0	N2—C30—C38—C39	179.0 (6)
C11—C1—C2—C3	−179.8 (9)	C28—C30—C38—C39	−1.1 (11)
C1—C2—C3—C4	0.0	C31—N3—C38—C30	−0.1 (7)
C2—C3—C4—C5	0.0	C31—N3—C38—C39	−178.7 (7)
C2—C3—C4—C12	−173.3 (9)	C30—C38—C39—C40	−0.7 (10)
C3—C4—C5—C6	0.0	N3—C38—C39—C40	177.7 (7)
C12—C4—C5—C6	172.9 (10)	C30—C38—C39—C41	−177.5 (7)
C3—C4—C5—C10	180.0	N3—C38—C39—C41	0.9 (12)
C12—C4—C5—C10	−7.1 (10)	C43—N4—C40—C39	1.2 (10)
C4—C5—C6—C7	180.0	Mn1—N4—C40—C39	−177.0 (5)
C10—C5—C6—C7	0.0	C43—N4—C40—C29	−179.6 (6)
C4—C5—C6—C1	0.0	Mn1—N4—C40—C29	2.2 (7)
C10—C5—C6—C1	180.0	C38—C39—C40—N4	−178.9 (6)
C2—C1—C6—C7	180.0	C41—C39—C40—N4	−1.8 (10)
C11—C1—C6—C7	−0.2 (9)	C38—C39—C40—C29	1.9 (9)
C2—C1—C6—C5	0.0	C41—C39—C40—C29	179.0 (6)
C11—C1—C6—C5	179.8 (9)	N1—C29—C40—N4	−1.0 (9)
C5—C6—C7—C8	0.0	C28—C29—C40—N4	179.3 (6)
C1—C6—C7—C8	180.0	N1—C29—C40—C39	178.2 (6)
C6—C7—C8—C9	0.0	C28—C29—C40—C39	−1.5 (10)
C7—C8—C9—C10	0.0	C40—C39—C41—C42	2.1 (10)
C8—C9—C10—C5	0.0	C38—C39—C41—C42	178.9 (7)
C6—C5—C10—C9	0.0	C39—C41—C42—C43	−1.9 (11)
C4—C5—C10—C9	180.0	C40—N4—C43—C42	−0.9 (10)
C6'—C1'—C2'—C3'	0.0	Mn1—N4—C43—C42	177.1 (5)
C11—C1'—C2'—C3'	176.5 (9)	C41—C42—C43—N4	1.3 (12)
C1'—C2'—C3'—C4'	0.0	C48—N5—C44—C45	0.8 (12)
C2'—C3'—C4'—C5'	0.0	Mn2—N5—C44—C45	−177.5 (7)
C2'—C3'—C4'—C12	−177.1 (8)	N5—C44—C45—C46	−2.2 (15)
C3'—C4'—C5'—C6'	0.0	C44—C45—C46—C47	1.9 (15)
C12—C4'—C5'—C6'	176.9 (8)	C45—C46—C47—C48	−0.5 (12)
C3'—C4'—C5'—C10'	180.0	C45—C46—C47—C49	−178.7 (8)
C12—C4'—C5'—C10'	−3.1 (8)	C44—N5—C48—C47	0.7 (10)
C4'—C5'—C6'—C7'	180.0	Mn2—N5—C48—C47	179.1 (5)
C10'—C5'—C6'—C7'	0.0	C44—N5—C48—C59	178.1 (6)
C4'—C5'—C6'—C1'	0.0	Mn2—N5—C48—C59	−3.4 (7)
C10'—C5'—C6'—C1'	180.0	C46—C47—C48—N5	−0.8 (11)
C2'—C1'—C6'—C7'	180.0	C49—C47—C48—N5	177.5 (6)
C11—C1'—C6'—C7'	3.6 (9)	C46—C47—C48—C59	−178.2 (7)
C2'—C1'—C6'—C5'	0.0	C49—C47—C48—C59	0.1 (10)
C11—C1'—C6'—C5'	−176.4 (9)	C50—N6—C49—C57	−0.9 (8)
C5'—C6'—C7'—C8'	0.0	C50—N6—C49—C47	−178.9 (7)
C1'—C6'—C7'—C8'	180.0	C48—C47—C49—N6	175.6 (7)
C6'—C7'—C8'—C9'	0.0	C46—C47—C49—N6	−6.2 (13)
C7'—C8'—C9'—C10'	0.0	C48—C47—C49—C57	−2.2 (10)
C8'—C9'—C10'—C5'	0.0	C46—C47—C49—C57	176.0 (7)
C4'—C5'—C10'—C9'	180.0	C49—N6—C50—N7	0.9 (8)
C6'—C5'—C10'—C9'	0.0	C49—N6—C50—C51	176.8 (7)
Mn2—O2—C11—O1	25.4 (13)	C57—N7—C50—N6	−0.6 (8)
Mn2—O2—C11—C1	−153.6 (6)	C57—N7—C50—C51	−176.5 (7)
Mn2—O2—C11—C1'	−158.4 (6)	N6—C50—C51—C52	5.0 (12)
Mn1^v^—O1—C11—O2	−116.3 (8)	N7—C50—C51—C52	−179.6 (7)
Mn1^v^—O1—C11—C1	62.7 (9)	N6—C50—C51—C56	−172.3 (7)
Mn1^v^—O1—C11—C1'	67.7 (9)	N7—C50—C51—C56	3.1 (12)
C2—C1—C11—O2	−128.5 (7)	C56—C51—C52—C53	1.0 (14)
C6—C1—C11—O2	51.7 (11)	C50—C51—C52—C53	−176.3 (8)
C2—C1—C11—O1	52.5 (9)	C51—C52—C53—C54	−2.7 (16)
C6—C1—C11—O1	−127.3 (7)	C52—C53—C54—C55	1.8 (17)
C2—C1—C11—C1'	−66 (7)	C53—C54—C55—C56	0.7 (16)
C6—C1—C11—C1'	114 (7)	C52—C51—C56—C55	1.5 (13)
C2'—C1'—C11—O2	59.2 (9)	C50—C51—C56—C55	178.7 (8)
C6'—C1'—C11—O2	−124.4 (8)	C54—C55—C56—C51	−2.4 (14)
C2'—C1'—C11—O1	−124.4 (7)	C50—N7—C57—C49	0.0 (7)
C6'—C1'—C11—O1	52.0 (11)	C50—N7—C57—C58	176.6 (7)
C2'—C1'—C11—C1	−60 (7)	N6—C49—C57—N7	0.6 (8)
C6'—C1'—C11—C1	116 (7)	C47—C49—C57—N7	178.8 (6)
Mn1^iv^—O4—C12—O3	−27.7 (11)	N6—C49—C57—C58	−176.3 (7)
Mn1^iv^—O4—C12—C4'	148.4 (6)	C47—C49—C57—C58	1.9 (11)
Mn1^iv^—O4—C12—C4	156.8 (6)	N7—C57—C58—C60	2.2 (13)
Mn2^vi^—O3—C12—O4	114.4 (8)	C49—C57—C58—C60	178.2 (7)
Mn2^vi^—O3—C12—C4'	−61.8 (8)	N7—C57—C58—C59	−175.3 (7)
Mn2^vi^—O3—C12—C4	−70.3 (9)	C49—C57—C58—C59	0.7 (11)
C5'—C4'—C12—O4	−53.8 (10)	C62—N8—C59—C58	0.3 (10)
C3'—C4'—C12—O4	123.1 (6)	Mn2—N8—C59—C58	175.9 (5)
C5'—C4'—C12—O3	122.6 (7)	C62—N8—C59—C48	180.0 (6)
C3'—C4'—C12—O3	−60.4 (7)	Mn2—N8—C59—C48	−4.4 (8)
C5'—C4'—C12—C4	−121 (5)	C60—C58—C59—N8	−0.9 (11)
C3'—C4'—C12—C4	56 (4)	C57—C58—C59—N8	176.9 (6)
C3—C4—C12—O4	−58.9 (8)	C60—C58—C59—C48	179.5 (7)
C5—C4—C12—O4	128.0 (7)	C57—C58—C59—C48	−2.8 (10)
C3—C4—C12—O3	125.4 (6)	N5—C48—C59—N8	5.3 (9)
C5—C4—C12—O3	−47.7 (11)	C47—C48—C59—N8	−177.2 (6)
C3—C4—C12—C4'	58 (4)	N5—C48—C59—C58	−175.0 (6)
C5—C4—C12—C4'	−116 (5)	C47—C48—C59—C58	2.4 (10)
Mn1—O6—C13—O5	−3.8 (7)	C59—C58—C60—C61	1.3 (12)
Mn1—O6—C13—C14	179.5 (5)	C57—C58—C60—C61	−176.2 (8)
Mn1—O5—C13—O6	3.4 (6)	C58—C60—C61—C62	−1.2 (13)
Mn1—O5—C13—C14	−179.9 (5)	C59—N8—C62—C61	−0.2 (12)
O6—C13—C14—C23	151.0 (6)	Mn2—N8—C62—C61	−175.3 (6)
O5—C13—C14—C23	−25.9 (8)	C60—C61—C62—N8	0.6 (13)
O6—C13—C14—C15	−32.5 (9)	C64—N9—C63—O9	−0.3 (4)
O5—C13—C14—C15	150.6 (6)	C65—N9—C63—O9	−179.8 (13)
C23—C14—C15—C16	177.6 (6)		

Symmetry codes: (i) *x*, *y*+1, *z*; (ii) *x*+1, *y*+1, *z*; (iii) *x*+1, *y*, *z*; (iv) *x*−1, *y*−1, *z*; (v) *x*, *y*−1, *z*; (vi) *x*−1, *y*, *z*.
